# The dose penumbra of a custom‐made shield used in hemibody skin electron irradiation

**DOI:** 10.1120/jacmp.v17i6.6367

**Published:** 2016-11-08

**Authors:** Charlotte I. Rivers, Ismail AlDahlawi, Iris Z. Wang, Anurag K. Singh, Matthew B. Podgorsak

**Affiliations:** ^1^ Department of Radiation Medicine Roswell Park Cancer Institute Buffalo NY USA; ^2^ Department of Physiology and Biophysics State University of New York at Buffalo Buffalo NY USA

**Keywords:** total skin electron irradiation, penumbra

## Abstract

We report our technique for hemibody skin electron irradiation with a custom‐made plywood shield. The technique is similar to our clinical total skin electron irradiation (TSEI), performed with a six‐pair dual field (Stanford technique) at an extended source‐to‐skin distance (SSD) of 377 cm, with the addition of a plywood shield placed at 50 cm from the patient. The shield is made of three layers of standard 5/8” thick plywood (total thickness of 4.75 cm) that are clamped securely on an adjustable‐height stand. Gafchromic EBT3 films were used in assessing the shield's transmission factor and the extent of the dose penumbra region for two different shield‐phantom gaps. The shield transmission factor was found to be about 10%. The width of the penumbra (80%‐to‐20% dose falloff) was measured to be 12 cm for a 50 cm shield‐phantom gap, and reduced slightly to 10 cm for a 35 cm shield‐phantom gap. *In vivo* dosimetry of a real case confirmed the expected shielded area dose.

PACS number(s): 87.53.Bn

## I. INTRODUCTION

Primary cutaneous T‐cell lymphoma (CTCL) is a subset of non‐Hodgkin's lymphoma confined to the skin at diagnosis. Staging and classification of CTCL is not always clear.^(1)^ According to records from the SEER database, over half of CTCLs are classified as mycosis fungoides,^(2)^ which is a diagnosis based on clinical and pathologic data. Staging of mycosis fungoides ranges from limited local disease to widespread patches, plaques and, eventually, even visceral organ involvement.^(1)^ While progression of the disease is generally slow, widespread skin involvement can be particularly bothersome for patients; in later stages, disease can be life‐threatening.

Treatment for mycosis fungoides ranges from topical skin therapies to systemic treatments. Topical options include corticosteroids, caryolysin, and PUVA (psoralen plus ultraviolet‐A); other topical chemotherapeutic agents may also be used, including carmustine and nitrogen mustard.^(3)^ Systemic chemotherapy is also an option, especially in cases with lymph node or widespread systemic disease.^(2)^ For many patients, disease remains refractory even after treatment with topical treatment and oral systemic agents. For these patients, radiation therapy has been found effective in inducing complete response.

Several techniques and strategies of radiation therapy are routinely used to treat mycosis fungoides. For patients with early stage disease limited to one location, local treatment with electrons can induce complete response and potentially even cure.^(4)^ For patients with more widespread disease, treatment with total skin electron irradiation (TSEI) has been shown to be effective in inducing complete response for prolonged time intervals. Several retrospective studies have shown that TSEI can result in complete clinical response in a majority of patients;^(5)^ while most patients eventually relapse, repeat TSEI or reirradiation with targeted electron therapy may be used and can produce repeat complete response.^(6)^

Delivery of total skin electron therapy is typically performed using the “Stanford technique”,^(7)^ in which the patient stands at least 300 cm from the radiation source, and moves through six standing positions (anterior, posterior, and four lateral oblique positions) in order to cover the entire skin surface. Areas of the patient receiving less than adequate dose may be boosted with supplemental local treatment (e.g., perineum, soles of feet). For patients unable to tolerate standing for long periods of time, an alternative method in which the patient lies on a table may also be used.^(8)^

While there are numerous techniques and options to treat mycosis fungoides patients with electron radiotherapy, both as an initial treatment as well as in the setting of reirradiation, there is little published data reporting on the use of the total skin technique to limited areas of the body. Müller‐Sievers et al.^(9)^ reported using a slit aperture made of 2 cm PMMA+3 mm aluminum plates as a shield in their rotational partial skin electron irradiation technique. Here we report our institution's experience treating mycosis fungoides limited to less than half of the skin surface with a “hemibody” skin electron technique. This technique serves to limit side effects by minimizing the area treated, as well as allowing for more aggressive reirradiation at the time of relapse.

## II. MATERIALS AND METHODS

### A. Irradiation technique and beam characteristics

Total skin electron irradiation (TSEI) in our clinic is performed with a six‐pair dual field technique (Stanford technique) at an extended source‐to‐skin distance (SSD) of 377 cm. The patient stands on an elevated platform with marks indicating the six different treatment positions 60° apart (RAO, AP, LAO, RPO, PA and LPO). Varian Trilogy (Varian Medical Systems, Palo Alto, CA) linear accelerator is used in a special procedure high dose rate total skin electron (HDTSe) mode with a nominal 6 MeV electron beam and a Lucite spoiler accessory for the TSEI treatments. Dual fields with gantry angles of $270^{\circ }\pm 22^{\circ }$ (i.e., 248° and 292°) are delivered for each patient position to achieve full longitudinal coverage and optimal dose uniformity. The dosimetric characteristics of our TSEI beam are shown in Table 1.

**Table 1:** Total skin electron irradiation (TSEI) beam dosimetric characteristics, for the combined six‐pair dual fields at an extended SSD of 377 cm

*Nominal Energy*	*6 MeV*
Depth of maximum dose ($d_{\text{max}}$)	0.3 cm
*R*_*50*_	1.32 cm in water
$E_{0}=2.33\;R_{50}$	3.08 MeV
$R_{p}$	2.21 cm in water
Bremsstrahlung photon dose background (at 3 cm depth)	∼2%
Dose Rate Output at $d_{\text{max}}$	24.75 cGy/100 MU for each field

**Figure 1:**
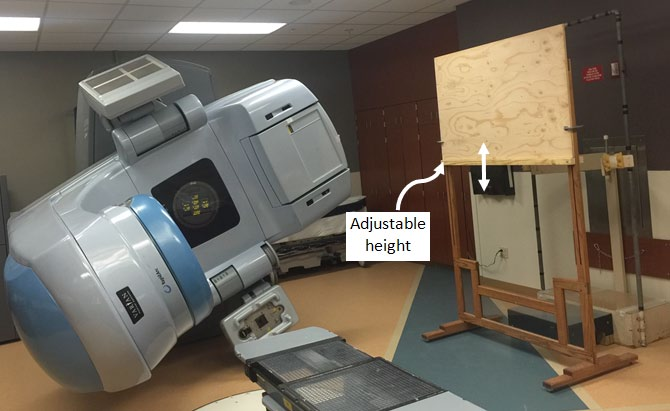
A photograph showing the custom‐design plywood shield on its stand. Linac gantry is angled at 248°. Also shown behind the shield is the patient stand.

In order to treat hemibody skin targets, a simple shield consisting of three rectangular layers (136 cm wide and 86 cm high) of standard 5/8” thick plywood (total thickness of 4.75 cm) is used. The plywood layers are clamped securely on a stand that is positioned 50 cm away from the patient (i.e., shortest shield‐patient gap we can use in clinical setup due to physical and safety restrictions). The position of the shield's inferior edge is adjustable to offer the physician the flexibility of matching the shield with the targeted skin area during the clinical treatment setup. Figure 1 shows the custom‐design shield setup.

### B. Transmission and penumbra measurement

Gafchromic EBT3 films (Ashland Inc., Wayne, NJ) were used to measure the custom‐designed shield transmission factor and assess the dose penumbra region under the shield for the TSEI electron beam. Film dose calibration was performed by irradiating pieces of films, from the same batch, to different doses ranging from 25 to 375 cGy, using a standard‐mode 6 MeV electron beam at the reference conditions ($d_{\text{max}},\;10\times 10\;\text{cm}$ field, and 100 cm SSD) in a Solid Water phantom (Gammex RMI, Middleton, WI). The optical density values of the irradiated films were analyzed 20 hrs postexposure using a 301 Densitometer (X‐rite Inc., Grand Rapids, MI). The characteristic H&D curve for the film was plotted and a fitting third order polynomial equation of dose vs. optical density was obtained.

**Figure 2:**
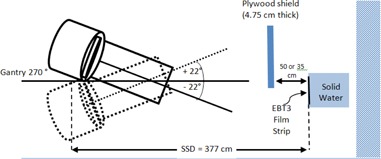
Schematic of the film transmission and penumbra measurements setup.

For the measurement, a 32 cm by 6 cm strip of Gafchromic EBT3 film from the same batch as the calibration films was used in determining the shield transmission and penumbra region for shield‐phantom gap of 50 cm, as well as for shield‐phantom gap of 35 cm to investigate the effect of reducing shield‐phantom gap on penumbra width. The film was placed on a surface of a Solid Water phantom facing the gantry with a 15 cm backscattering material, as shown in Fig. 2. The irradiations were performed for the three dose‐contributing dual fields (i.e., AP, RAO and LAO) in the HDTSe mode. Identical fields used to irradiate the posterior region of a patient were not used in penumbra assessment as these would not contribute to the surface dose on patient's anterior region. The films were analyzed using the 301 Densitometer 20 hrs postirradiation to be consistent with the calibration postprocessing period.

### C. Verification of delivery dose

To verify the dose delivered for hemibody skin radiation using our custom‐designed shield in a real patient treatment, *in vivo* dosimetry using Gafchromic EBT3 films were performed. The shield was placed at 50 cm away from the patient. The intention was to target the lower‐body skin to a total dose of 9 Gy in 5 fractions (i.e., 180 cGy per fraction). Eye shields were clinically deemed unnecessarily due to the low prescribed dose and favoring patient comfort during positioning and treatment. Film pieces were taped on the patient skin to measure the dose received during the first two fractions. The films were placed on the patient forehead and upper body (under shielded region); and also at the level of pelvic area — about 8 cm below shield edge, left anterior thigh, and left ankle to verify the delivered dose. Films were analyzed 20 hrs postexposure using a 301 Densitometer.

## III. RESULTS AND DISCUSSION

### A. Transmission and penumbra measurements

**Figure 3:**
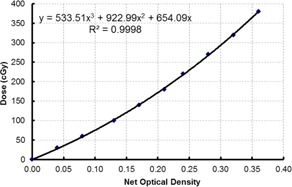
Gafchromic EBT3 film characteristic curve.

The film characteristic curve, plotted in Fig. 3, was used to convert film optical density reading to dose. The transmission factor of the custom‐design plywood shield was determined by normalizing the dose under the shield to the dose at the open region, and found to be 10%. Although adding another layer of plywood (5/8” thick) will further reduce the shield transmission factor, this is not practical in our case as the added plywood weight on the stand holder would create instability in stand positioning. Also, considering the low dose prescribed (9 Gy in 5 fractions), we were comfortable with the amount of transmission through the plywood. Gamble et al.^(10)^ also mentioned the use of a plywood shield in their total skin electron beam technique with a similar attenuation factor of 90%, though the thickness or placement of the plywood was not detailed in their article.

**Figure 4:**
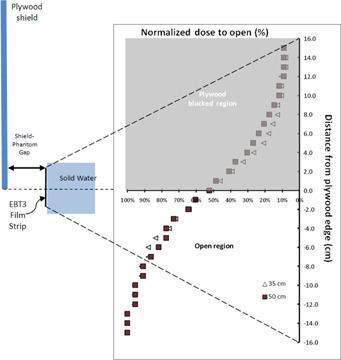
Relative dose profile versus distance from the shield edge (positive distance is under shielded region) measured with Gafchromic EBT3 films for shield‐phantom gaps of 50 cm (squares) and 35 cm (triangles).

The film measurements also revealed a wide penumbra (from 80% to 20% dose falloff) centered at the shield edge, as seen in Fig. 4. The penumbra width is about 12 cm for shield‐phantom gap of 50 cm. Reducing the air gap between the shield and the patient (i.e., bringing the shield closer to the patient) to 35 cm slightly reduced the penumbra width to 10 cm; however physical limitations and safety considerations should be taken into account when setting up a small shield‐patient gap. Dose enhancement around shield edge due to electron scattering should also be considered. The Müller‐Sievers study^(9)^ concluded that increased distance between shield and phantom minimized the dose fluctuations around shield edges, and that for a distance of 25 cm or larger, homogenous dose distribution around the edge was noticed. Our shortest experimental shield‐phantom gap of 35 cm did not show increase in dose fluctuations around the shield edge at the patient surface level, in agreement with the findings by Müller‐Sievers and colleagues.

### B. Verification of delivery dose

The results of film measurements during the first two fractions of treatment are shown in Table 2. Measurements of doses in shielded sites (i.e., the forehead and upper body) are consistent with the transmission factor results at a dose 10% of prescription. The doses delivered to the lower‐body skin are about 14%–18% higher than the prescribed 180 cGy dose, which exceeds the typically desired dose uniformity of 10% in total skin electron irradiation (TSEI).^(11)^ This might be explained by the large anatomy of this particular patient. Our patient also had to position himself more anteriorly in order to balance his body weight. The large dose variation at the ankle (2% higher than prescription in the first fraction and 26% higher in the second fraction) is probably due to the inconsistency in positioning, similar to the cases of total skin electron irradiation (no‐shield) delivered in our clinic. Several studies^12^, ^13^, ^14^, ^15^, ^16^ have shown large variation range of dose to different body parts in total skin irradiation with six‐pair dual field technique as well.

**Table 2:** Results of *in vivo* dosimetry measurements for different body sites for two fractions of 180 cGy/fractions

	*Film Location*	*Net OD*	*Dose (cGy)*	*Average Dose (cGy)*	*Ratio to Prescription*
Under Shield	Forehead	0.03 – 0.03	20.5 – 20.5	20.5	0.11
	Upper Body	0.02 – 0.03	13.5 – 20.5	17.6	0.10
Open	Lower Body (at level of pelvis)	0.22 – 0.24	194.3 – 217.5	205.8	1.14
	Left anterior thigh	0.23 – 0.24	205.8 – 217.5	212.8	1.18
	Left ankle	0.21 – 0.26	183.0 – 241.8	227.1	1.26

## IV. CONCLUSIONS

We have presented our simple custom‐designed shield for a hemibody skin electron irradiation we use in our clinic. The shield is made of three layers of standard 5/8” thick plywood (total thickness of 4.75 cm) clamped securely on an adjustable‐height stand with a transmission factor of 10%, and about a 12 cm wide penumbra (distance of 80%‐to‐20% dose falloff) around the shield edge for a shield‐patient gap of 50 cm. *In vivo* dosimetry of a real case confirmed the expected dose of the area behind the plywood shield. We have treated several hemibody skin patients with this custom‐made plywood shield; the current patient measurements are representative of these for other patients as well. Our hemibody skin electron irradiation technique serves to limit side effects by minimizing the area treated, and allows for reirradiation in case of relapse.

## COPYRIGHT

This work is licensed under a Creative Commons Attribution 3.0 Unported License.
